# Interleukin-1β Induces Tissue Factor Expression in A549 Cells via EGFR-Dependent and -Independent Mechanisms

**DOI:** 10.3390/ijms22126606

**Published:** 2021-06-21

**Authors:** Tobias Mechelke, Felix Wittig, Robert Ramer, Burkhard Hinz

**Affiliations:** Institute of Pharmacology and Toxicology, Rostock University Medical Center, Schillingallee 70, 18057 Rostock, Germany; tobias.mechelke@uni-rostock.de (T.M.); felix.wittig@med.uni-rostock.de (F.W.); robert.ramer@med.uni-rostock.de (R.R.)

**Keywords:** interleukin-1β, tissue factor, epidermal growth factor receptor, mitogen-activated protein kinases, erlotinib, lung cancer cells

## Abstract

Tissue factor (TF) plays an important role in the progression and angiogenesis of tumor cells. The present study investigated the mechanism of interleukin-1β (IL-1β)-induced TF expression in A549 lung cancer cells. Based on mRNA and protein analyses, including appropriate inhibitor experiments, IL-1β was shown to induce TF expression in a time-dependent manner, mediated by IL-1 receptor-dependent phosphorylation of the mitogen-activated protein kinases (MAPK) p38, p42/44 and c-jun N-terminal kinase (JNK), as well as the Src kinase and the epidermal growth factor receptor (EGFR). Thereby, inhibition of EGFR transactivation by the Src inhibitor PP1 or direct EGFR inhibition by the EGFR tyrosine kinase inhibitor (TKI) erlotinib led to a reduction of IL-1β-induced TF expression and to a suppression of p42/44 MAPK and EGFR activation, while IL-1β-induced p38 MAPK and JNK activation remained unchanged. A knockdown of EGFR by siRNA was associated with decreased IL-1β-mediated p42/44 MAPK activation, which was no longer inhibitable by erlotinib. Concentration-dependent inhibition of IL-1β-induced TF expression was also observed in the presence of gefitinib and afatinib, two other EGFR TKIs. In summary, our results suggest that IL-1β leads to increased TF formation in lung cancer cells via both Src/EGFR/p42/44 MAPK-dependent and EGFR-independent signaling pathways, with the latter mediated via p38 MAPK and JNK.

## 1. Introduction

The tissue factor represents a 47-kDa transmembrane glycoprotein, which is considered the primary initiator of blood coagulation [1,2,3]. In this process, the protein, which is mainly expressed subendothelially, comes into contact with coagulation factor VII in endothelial damage, whereby the resulting complex of TF and activated factor VII (FVIIa) leads to the activation of factor X (FX) and thus to the formation of blood clotting inducing thrombin. In addition, TF plays an important role in tumor growth, angiogenesis, thrombogenicity and metastasis, whereby a high level of tumor TF expression is associated with a poor prognosis in a number of cancer types (for review see [4,5,6,7]). With regard to the molecular mechanism, it has been shown, for example, that TF in the form of the binary TF-FVIIa and the ternary TF-FVIIa-FXa complex leads to an induction of cancer cell proliferation and migration via activation of the protease-activated receptor (PAR)-2 [8,9]. Together, these properties make TF a potential target for cancer therapy [6,7].

With regard to lung cancer, a significant relationship between TF expression and microvascular density has been described in non-small cell lung cancer (NSCLC), suggesting a proangiogenic function of TF [10]. Later, it was shown that TF is significantly raised in NSCLC patients and that elevated TF expression is associated with increased blood thrombogenicity [11] and may play a significant role in metastasis [12]. Furthermore, one study suggested that TF gene expression could be used as a prognostic marker in NSCLC [13]. 

In cancer, TF is upregulated by a number of external stimuli and oncogenic mutations. This includes activation of TF by the oncogenic epidermal growth factor receptor (EGFR), with corresponding studies on glioma [14,15,16], epidermoid [14,17,18], colorectal [18], breast [19] and endometrial carcinoma cells [20]. Transactivation of EGFR has been described for the proinflammatory cytokine interleukin (IL)-1β in different cells [21,22,23,24,25,26,27], including an NSCLC cell line [23], which raises the question of the possible involvement of EGFR even in IL-1β-induced TF expression. Surprisingly, IL-1β, a profound inducer of TF expression in endothelial cells [28,29,30,31] and monocytes [32], has not yet been investigated in detail with regard to the molecular mechanisms of a corresponding regulation in tumor cells. However, appropriate analyses seem to be advisable, since inflammation is an important component of tumor progression [33], and elevated plasma levels of IL-1β have been found in patients with both small cell lung cancer and NSCLC [34]. 

The present study therefore investigates the effect of IL-1β on TF expression in the NSCLC cell line A549, an established cellular system for analyses of IL-1β effects [23,35,36,37,38], and the underlying mechanisms. Thereby, the existence of an EGFR-independent mechanism via stimulation of p38 mitogen-activated protein kinase (MAPK) and c-jun N-terminal kinase (JNK) as well as an Src/EGFR-dependent pathway involving downstream activation of p42/44 MAPK was demonstrated. 

## 2. Results

### 2.1. IL-1β Induces TF Expression in A549 Lung Cancer Cells

**Figure 1:**
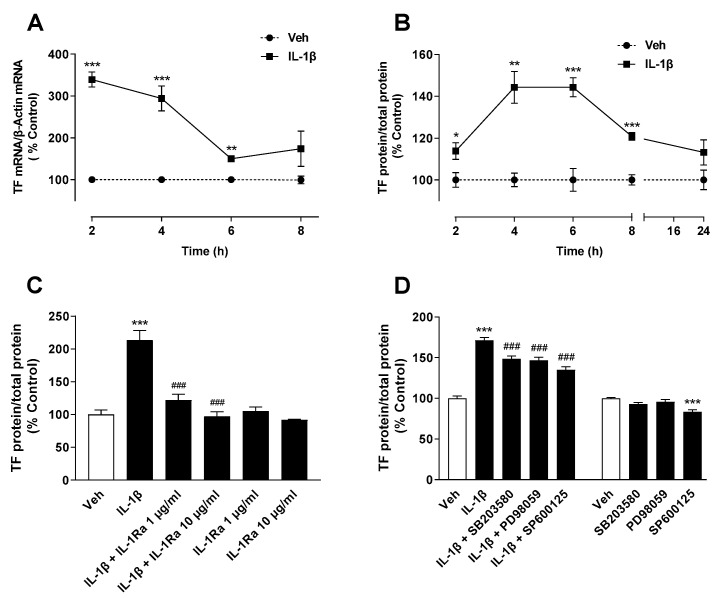
IL-1β induces TF expression in A549 cells, which is completely abolished by IL-1 receptor antagonism and partially by inhibition of p38 MAPK, p42/44 MAPK and JNK. Time course of IL-1β-induced TF mRNA (**A**) and TF protein (**B**). Effect of IL-1Ra (**C**, at indicated concentrations) and SB203580, PD98059 and SP600125 (**D**, all at 10 µM) on IL-1β-induced or basal TF protein. The cells were incubated in the presence or absence of IL-1β (10 ng/mL) for the indicated periods (**A**,**B**) or preincubated with the respective inhibitor for 2 h (**C**) or 1 h (**D**) and then coincubated with IL-1β or vehicle for 6 h. All percentage values shown refer to the respective vehicle control, which was set to 100%. The data represent mean values ± SEM of n = 4 (**A**,**B**), n = 3 (**C**) or n = 8 (**D**) experiments per group. * *p* ≤ 0.05, ** *p* ≤ 0.01, *** *p* ≤ 0.001 vs. corresponding vehicle control; ### *p* ≤ 0.001 vs. IL-1β; Student’s *t* test (**A**,**B**), one-way ANOVA with Bonferroni’s post hoc test (**C**,**D**, left columns) or one-way ANOVA with Dunnett’s post hoc test (**D**, right columns).

In initial experiments, the time dependence of the TF induction by IL-1β was investigated. Corresponding kinetics experiments (Figure 1A,B) showed expression maxima after 2 h (mRNA) and 4 to 6 h (protein), respectively. Therefore, further experiments were performed at the TF protein level using a 6-h incubation with IL-1β.

### 2.2. TF Expression Induced by IL-1β Is Mediated via IL-1 Receptor-Dependent Activation of MAP Kinases p38, p42/44 and JNK

In order to obtain the first indications of the mechanism of TF induction, inhibitor experiments with the IL-1 receptor antagonist (IL-1Ra) and with inhibitors of MAPKs were performed. Thereby, IL-1Ra induced a complete blockade of IL-1β-induced TF formation (Figure 1C), while inhibitors of p38 MAPK (SB203580) and JNK (SP600125) as well as an inhibitor of p42/44 MAPK activation (PD98059) induced partial but significant suppression of TF protein production (Figure 1D). Remarkably, viability checks for all of the groups shown in Figure 1C,D showed no evidence of a significant change in cellular viability by the individual substances and substance combinations within the incubation period used for TF protein measurement (data not shown).

**Figure 2:**
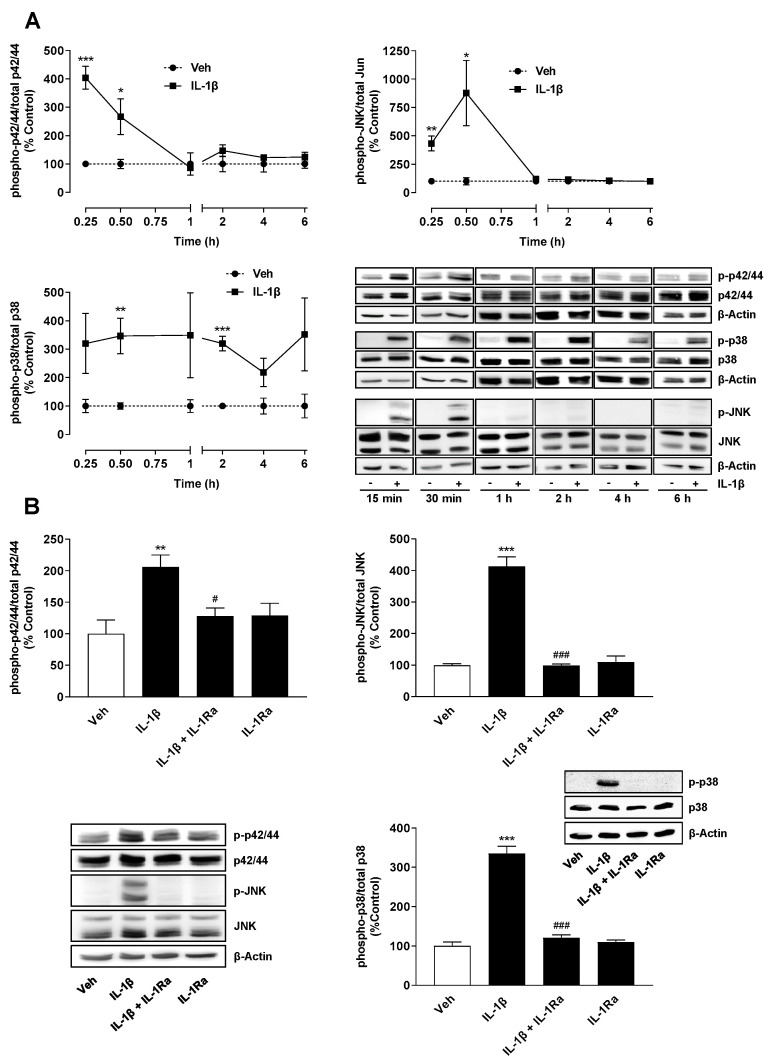
IL-1 receptor-dependent activation of p42/44 MAPK, JNK and p38 MAPK. Time course of IL-1β-induced activation of p42/44 MAPK, JNK and p38 MAPK (**A**). Effect of IL-1Ra (10 µg/mL) on IL-1β-induced or basal p42/44 MAPK, JNK and p38 MAPK activation (**B**). The cells were incubated in the presence or absence of IL-1β (10 ng/mL) for the specified time periods (**A**) or preincubated with IL-1Ra for 2 h and then coincubated with IL-1β or vehicle for 30 min (**B**). All percentage values shown refer to the respective vehicle control, which was set to 100%. The values shown in the histograms are based on densitometric analyses of blots, whereby the phosphorylated forms were normalized to the respective total form. In the labelling of the MAPK blots (**A**,**B**), the prefix “p-” stands for “phospho-”. The blots shown are representative. In (**A**), the same β-actin blots are shown for the 1-, 2-, 4- and 6-h values of the p42/44 and p38 MAPK analyses, because the analyses of the same lanes were shown here as representative blots. The data are mean values ± SEM of n = 4 (**A**,**B**, upper right), n = 7–8 (**B**, upper left) or n = 3 (**B**, lower right) experiments per group. * *p* ≤ 0.05, ** *p* ≤ 0.01, *** *p* ≤ 0.001 vs. vehicle control; # *p* ≤ 0.05, ### *p* ≤ 0.001 vs. IL-1β; Student’s *t* test (**A**) or one-way ANOVA with Bonferroni’s post hoc test (**B**).

An analysis of the phosphorylated forms of the mentioned kinases (Figure 2A) showed a time-dependent induction with activation maxima after 15 min (p42/44 MAPK), 30 min (p38 MAPK) and 30 min (JNK), respectively. Further experiments confirmed a complete inhibition of IL-1β-induced phosphorylation of all three MAPKs by IL-1Ra (Figure 2B).

### 2.3. TF Expression Induced by IL-1β Is Mediated by an IL-1 Receptor-Dependent Activation of EGFR

**Figure 3:**
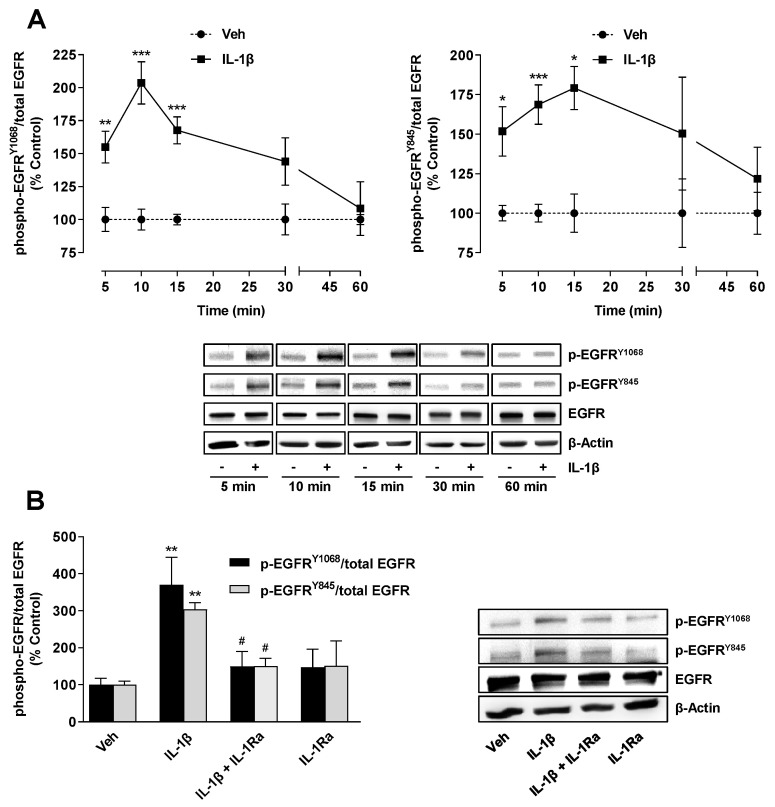
TF expression induced by IL-1β is mediated by an IL-1 receptor-dependent activation of EGFR. Time course of IL-1β-induced EGFR activation at Tyr1068 and Tyr845 (**A**). Effect of IL-1Ra (10 µg/mL) on IL-1β-induced or basal EGFR activation at Tyr1068 and Tyr845 (**B**). The cells were incubated in the presence or absence of IL-1β (10 ng/mL) for the indicated time periods (**A**) or preincubated with IL-1Ra for 2 h and then coincubated with IL-1β for 10 min (**B**). All percentage values shown refer to the respective vehicle control, which was set to 100%. The values shown in the graphs are based on densitometric analyses of blots, whereby the phosphorylated forms were normalized to the respective total form. In the labelling of the EGFR blots (**A**,**B**) and in the legend of the histogram (**B**), the prefix “p-” stands for “phospho-”. The blots shown are representative. The data are mean values ± SEM of n = 3–10 (**A**, left), n = 3–7 (**A**, right) or n = 4 (**B**) experiments per group. * *p* ≤ 0.05, ** *p* ≤ 0.01, *** *p* ≤ 0.001 vs. corresponding vehicle control; # *p* ≤ 0.05 vs. IL-1β; Student’s *t* test (**A**) or one-way ANOVA with Bonferroni’s post hoc test (**B**).

On the basis of literature findings on the transactivation of EGFR by IL-1β [21,22,23,24,25,26,27] and the involvement of EGFR in TF expression in other cancer cell types [14,15,16,17,18,19], a Western blot analysis of EGFR phosphorylation at Tyr1068 and Tyr845 in IL-1β-stimulated A549 cells was performed (Figure 3A). Thereby, a time-dependent induction of phosphorylation with maxima after 10 min (Tyr1068) and 15 min (Tyr845) was shown. The IL-1β-induced EGFR activation was likewise almost completely eliminated in the presence of IL-1Ra (Figure 3B).

**Figure 4:**
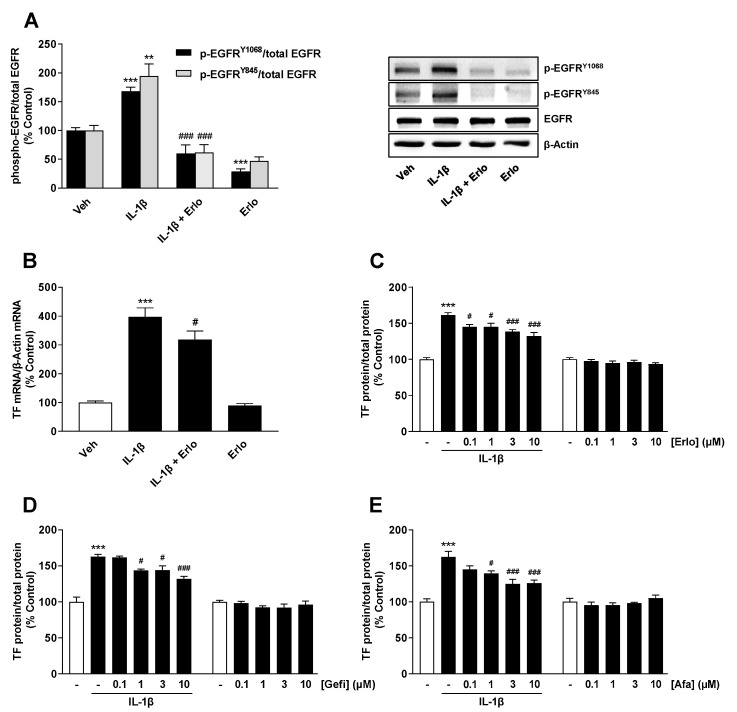
Therapeutically used EGFR TKIs suppress TF expression in A549 cells. (**A**) Effect of erlotinib (Erlo) at 3 µM on IL-1β-induced or basal EGFR activation at Tyr1068 and Tyr845. Effect of erlotinib on IL-1β-induced or basal TF mRNA (**B**, at 3 µM) and protein (**C**, at the concentrations indicated). (**D**,**E**) Effect of gefitinib (Gefi) and afatinib (Afa) on IL-1β-induced or basal TF protein at the concentrations indicated. The cells were incubated in the presence or absence of IL-1β (10 ng/mL) for 10 min (**A**), 2 h (**B**) or 6 h (**C**–**E**). EGFR TKIs were added to the cells immediately before IL-1β. All percentage values shown refer to the respective vehicle control, which was set to 100%. The values shown in the first histogram (**A**) are based on densitometric analyses of blots, whereby the phosphorylated forms were normalized to the respective total form. In the labelling of the EGFR blots (**A**) and in the legend of the histogram (**A**), the prefix “p-” stands for “phospho-”. The blots shown are representative. All data are mean values ± SEM of n = 4 (**A**,**D**,**E**), n = 11–12 (**B**) or n = 8 (**C**) experiments per group. ** *p* ≤ 0.01, *** *p* ≤ 0.001 vs. corresponding vehicle control; # *p* ≤ 0.05, ### *p* ≤ 0.001 vs. IL-1β; one-way ANOVA with Bonferroni’s post hoc test. A statistically significant influence of EGFR TKIs on basal TF expression (**C**–**E**, respective columns shown on the right) was excluded by one-way ANOVA with Dunnett’s post hoc test.

Subsequent experiments were performed with the EGFR tyrosine kinase inhibitor (TKI) erlotinib. The efficiency of erlotinib as an EGFR inhibitor in our system was confirmed by Western blot analyses, which showed complete inhibition of IL-1β-induced EGFR phosphorylation by erlotinib (Figure 4A). Erlotinib showed a significant, albeit partial, inhibition of IL-1β-induced TF expression at the mRNA (Figure 4B) and protein level (Figure 4C). To exclude off-target effects, two further EGFR TKIs, gefitinib (Figure 4D) and afatinib (Figure 4E), were tested, which also proved to be inhibitors of IL-1β-induced TF expression. In the case of afatinib, the inhibitory effect on TF expression appeared to reach a plateau above a concentration of 3 µM. For the three EGFR TKIs tested, no significant cytotoxic effect could be detected within the incubation time of 6 h relevant for TF measurement when using the incubation setups shown in Figure 4C–E, both in the presence and absence of IL-1β (data not shown).

### 2.4. Src Is Involved in IL-1β-Mediated Transactivation of EGFR Leading to TF Upregulation

**Figure 5:**
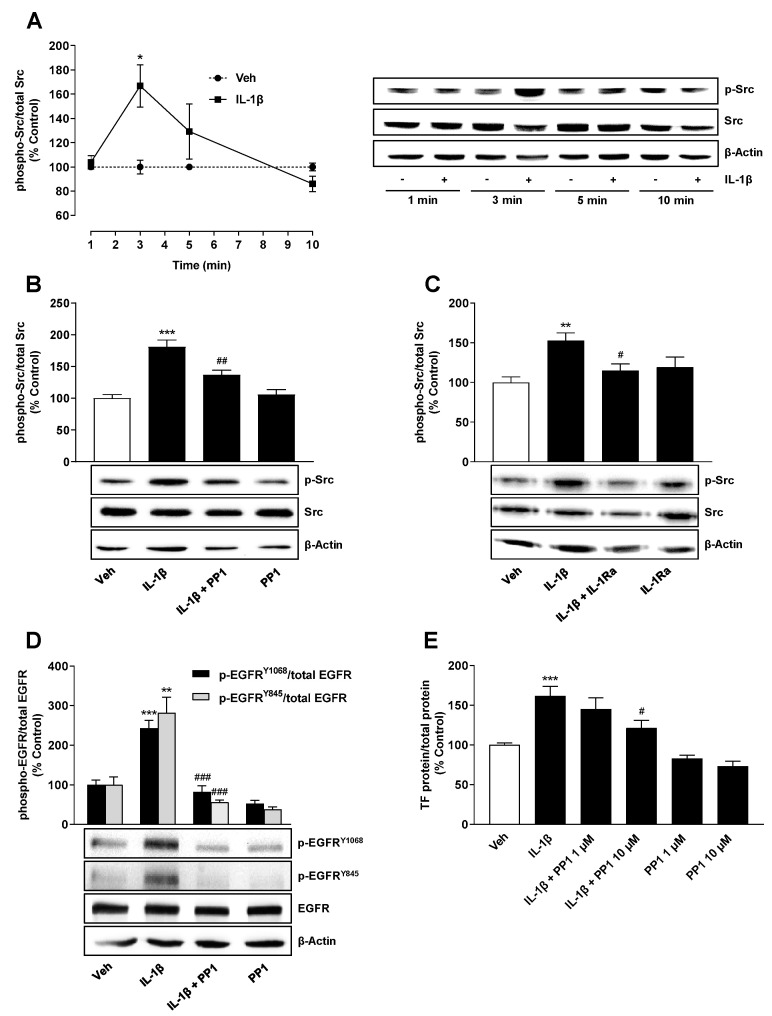
Src phosphorylation induced by IL-1β confers EGFR activation and subsequent TF expression. Time course of IL-1β-induced activation of Src (**A**). Effect of the Src inhibitor PP1 (**B**) and IL-1Ra (**C**) on IL-1β-induced Src activation. Impact of PP1 on EGFR activation at Tyr1068 and Tyr845 (**D**) and TF protein formation (**E**). The cells were incubated in the presence or absence of IL-1β (10 ng/mL) for the specified time periods (**A**), 3 min (**B**,**C**), 10 min (**D**) or 6 h (**E**). The cells were preincubated with PP1 for 1 h at 10 µM (**B**,**D**) or at the indicated concentrations (**E**). In case of IL-1Ra (**C**), cells were incubated with the receptor antagonist at 10 µg/mL for 2 h and then coincubated with IL-1β or vehicle for the times indicated above. All percentage values shown refer to the respective vehicle control, which was set to 100%. The values shown in the graphs **A**–**D** are based on densitometric analyses of blots, whereby the phosphorylated forms were normalized to the respective total form. In the labelling of the Src (**A**–**C**) and EGFR blots (**D**) as well as in the legend (**D**), the prefix “p-” stands for “phospho-”. The blots shown are representative. All data are mean values ± SEM of n = 3 (**A**), n = 5 (**B**), n = 7 (**C**), n = 3 (**D**, p-EGFR^Y845^), n = 4 (**D**, p-EGFR^Y1068^) or n = 7–8 (**E**) experiments per group. * *p* ≤ 0.05, ** *p* ≤ 0.01, *** *p* ≤ 0.001 vs. vehicle control; # *p* ≤ 0.05, ## *p* ≤ 0.01, ### *p* ≤ 0.001 vs. IL-1β; Student’s *t* test (**A**) or one-way ANOVA with Bonferroni’s post hoc test (**B**–**E**).

Src, a nonreceptor protein tyrosine kinase, has been reported to be a mediator of EGFR transactivation in the absence of EGFR ligands (for review see [39,40]). To investigate whether Src mediates IL-1β-induced EGFR activation, the phosphorylation of this kinase was first addressed in time course experiments. According to Figure 5A, the incubation of A549 cells with IL-1β led to a time-dependent induction of the phosphorylated form of Src with a respective significant increase after a 3-min incubation.

Further analysis of this induction showed that both the Src inhibitor PP1 (Figure 5B), which, in studies by other authors, inhibited EGFR transactivation [23,41,42,43], and IL-1Ra (Figure 5C) led to a significant inhibition of IL-1β-induced Src phosphorylation. These results confirmed the efficiency of PP1 as Src inhibitor in our system and, on the other hand, revealed that IL-1β induces an IL-1 receptor-dependent activation of Src.

The role of Src in EGFR transactivation was finally confirmed by the complete reversal of IL-1β-induced EGFR phosphorylation in the presence of PP1, both at Tyr1068 and Tyr845 (Figure 5D). PP1 consequently led to a significant inhibition of IL-1β-induced TF expression (Figure 5E). Using the same incubation protocol in terms of groups and times as in Figure 5E, no inhibition of viability of A549 cells could be detected for PP1 either in the absence or presence of IL-1β (data not shown).

### 2.5. IL-1β Induces TF Expression via Both a Src/EGFR/p42/44 MAPK-Dependent and a Src/EGFR-Independent Pathway Mediated by Activation of p38 MAPK and JNK

**Figure 6:**
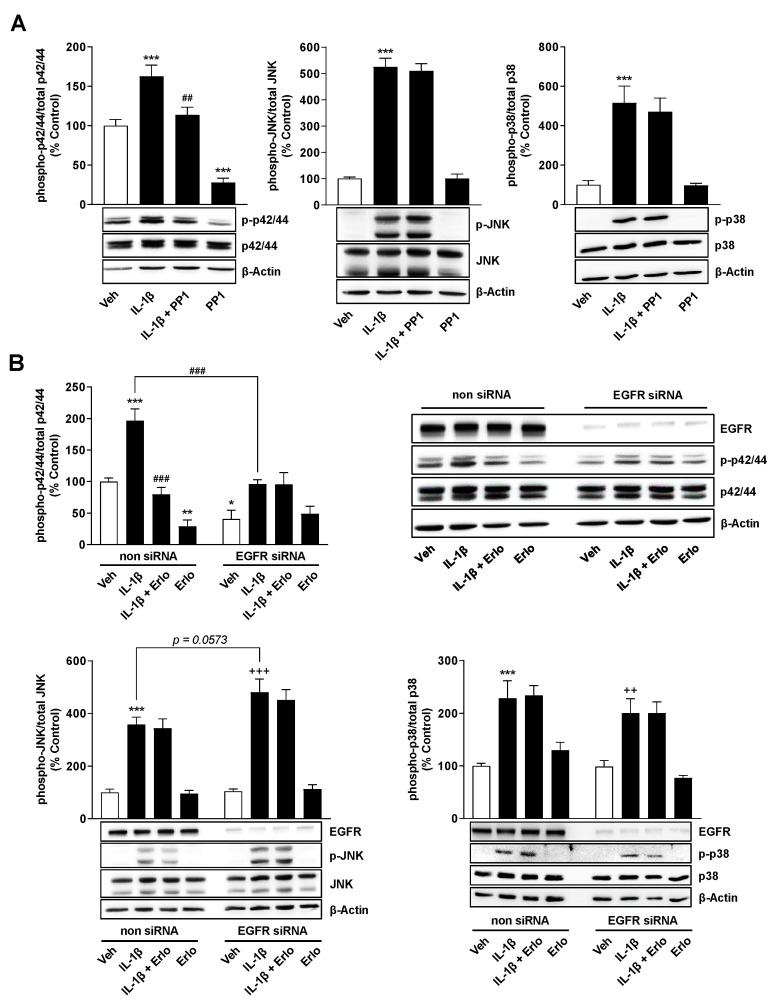
The Src kinase inhibitor PP1 and the EGFR TKI erlotinib inhibit IL-1β-induced p42/44 MAPK, but not JNK and p38 MAPK activation. (**A**) Effect of PP1 at 10 µM on IL-1β-induced or basal p42/44 MAPK, JNK and p38 MAPK activation in A549 cells. (**B**) Effect of erlotinib (Erlo) at 3 µM on IL-1β-induced or basal p42/44 MAPK, JNK and p38 MAPK activation in cells transfected with nonsilencing siRNA (non siRNA; negative control siRNA) or EGFR siRNA at 10 nM each. In (**A**), the cells were preincubated with PP1 for 1 h followed by the addition of IL-1β (10 ng/mL) or vehicle and coincubation for 30 min. In (**B**), erlotinib was added immediately before IL-1β or vehicle followed by coincubation for 30 min. All percentage values shown refer to the respective vehicle control, which was set to 100%. The values shown in the histograms are based on densitometric analyses of blots, whereby the phosphorylated forms were normalized to the respective total form. In the labelling of the MAPK blots (**A**,**B**) the prefix “p-” stands for “phospho-”. The blots shown are representative. The data are mean values ± SEM of n = 10 (**A**, left), n = 4 (**A**, middle, **B**) or n = 5 (**A**, right) experiments per group. * *p* ≤ 0.05, ** *p* ≤ 0.01, *** *p* ≤ 0.001 vs. vehicle control (**A**) or vehicle control of non siRNA transfection (**B**); ## *p* ≤ 0.01, ### *p* ≤ 0.001 vs. IL-1β (**A**) or IL-1β in non siRNA-transfected cells (**B**); ++ *p* ≤ 0.01, +++ *p* ≤ 0.001 vs. vehicle control of EGFR siRNA transfection; one-way ANOVA with Bonferroni’s post hoc test.

With regard to the involvement of the three MAPKs p42/44, JNK and p38 in the IL-1β-induced TF formation shown above (Figure 1 and Figure 2), the next step was to focus on the involvement of the three kinases as potential downstream targets of EGFR activation (Figure 6).

Thereby, as shown in Figure 6A, a significant inhibition of phosphorylation of p42/44 MAPK, but not of activation of p38 MAPK and JNK, could be demonstrated in the presence of PP1, suggesting the parallel existence of Src/EGFR-dependent and -independent signaling pathways in IL-1β-induced TF expression. To confirm these results, further experiments with EGFR TKI erlotinib, which acts downstream to PP1, were performed in cells transfected with nonsilencing or EGFR siRNA. Erlotinib induced the same activation pattern as PP1 in cells transfected with nonsilencing siRNA by completely inhibiting IL-1β-induced p42/44 MAPK activation while being ineffective against JNK and p38 MAPK phosphorylation (Figure 6B). On the other hand, EGFR knockdown was associated with significantly lower IL-1β-induced p42/44 MAPK activation, which was not inhibited by erlotinib under these circumstances (Figure 6B). Interestingly, EGFR knockdown led to an increase in JNK activity by IL-1β compared to the IL-1β-stimulated group treated with nonsilencing siRNA, with the difference (*p* = 0.0573) only just missing the significance threshold (Figure 6B).

## 3. Discussion

**Figure 7:**
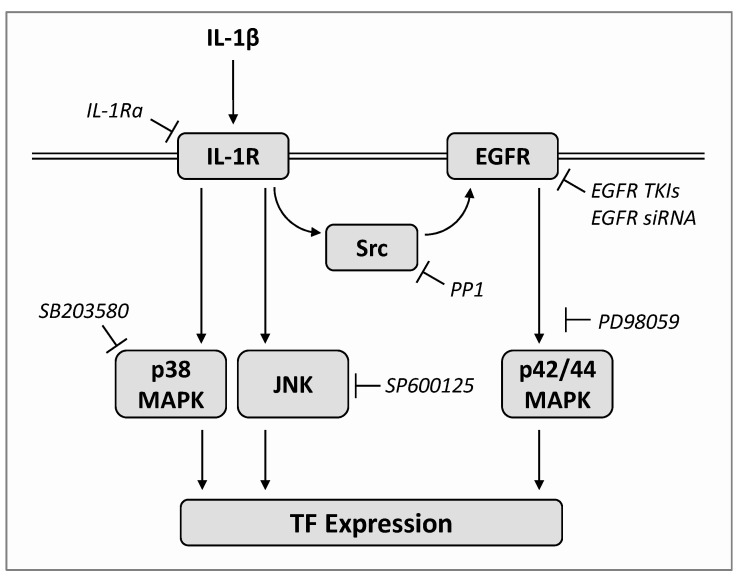
Proposed mechanism underlying IL-1β-induced TF expression in A549 cells. IL-1β induces IL-1 receptor (IL-1R)-dependent phosphorylation of p42/44 MAPK, JNK, p38 MAPK, Src and EGFR. While the activation of JNK and p38 MAPK is independent of the activation of Src and EGFR, p42/44 MAPK activation represents a Src/EGFR-dependent event. TF expression is therefore triggered by both Src/EGFR/p42/44 MAPK-dependent and Src/EGFR-independent signaling, the latter being mediated via p38 MAPK and JNK. The arrows mark the effect triggered by IL-1β in the absence of the indicated inhibitors, which are shown in italics at the respective position of their inhibitory effect in the cascade.

Research in recent years has shown that TF plays an important role in the progression and angiogenesis of tumor cells, making TF a potential target for cancer therapy. In the present study, it was shown that the proinflammatory cytokine IL-1β, which is abundantly produced in lung cancer [10], leads to increased TF formation in lung cancer cells via both Src/EGFR/p42/44 MAPK-dependent and EGFR-independent signaling pathways, the latter being mediated via p38 MAPK and JNK (Figure 7). The inhibition of IL-1β-induced TF expression proven in this context for various EGFR TKIs and IL-1 receptor antagonist (IL-1Ra) underlines the fact that TF-inhibiting substances are already currently used therapeutically with these drugs.

There is a body of experimental evidence supporting the above conclusions. First, based on inhibitor experiments and Western blot analyses, it could be shown that IL-1β-induced TF expression is mediated by IL-1 receptor-dependent phosphorylation of p38 MAPK, p42/44 MAPK and JNK, the nonreceptor protein tyrosine kinase Src and EGFR. Second, and consistent with this, the inhibition of EGFR transactivation by the Src inhibitor PP1 or direct EGFR inhibition by erlotinib resulted in the suppression of IL-1β-induced TF expression, p42/44 MAPK and EGFR activation, while IL-1β-induced p38 MAPK and JNK activation remained unchanged. Thirdly, siRNA-mediated EGFR knockdown was associated with a decreased IL-1β-mediated p42/44 MAPK activation that was no longer inhibitable by erlotinib.

EGFR transactivation by IL-1β is supported by a number of other studies [21,22,23,24,25,26,27]. In the case of IL-1β-mediated TF formation addressed in this study, the activation of the nonreceptor protein tyrosine kinase Src, which mediates EGFR activation in the absence of its ligand EGF (for review see [39,40]), seems to play an essential role. In accordance with this, IL-1β-induced TF expression and upstream activation of EGFR and p42/44 MAPK were completely suppressed by the Src inhibitor PP1. As a molecular mechanism of this transactivation, a conformational change of the EGFR kinase domain mediated by Src is postulated with the consequence of phosphorylation of thus accessible tyrosine residues [40]. EGFR can be phosphorylated by Src at multiple sites, most notably Tyr845 [44], but also Tyr891, Tyr920 and Tyr1101 [42]. The phosphorylation of Tyr1068, which, in addition to Tyr845, was suppressed by the Src inhibitor PP1 in the present study, is described controversially in the literature. In accordance with our data, Tyr1068 has also been revealed to be a Src substrate in glioblastoma cells [45]. In another study with the NSCLC cell line HCC827, a reduced phosphorylation of EGFR at Tyr845 but not at Tyr1068 was found after small hairpin RNA-mediated Src depletion [46]. For IL-1β, Src-dependent EGFR activation was also described with respect to IL-1β-induced matrix metalloproteinase-9 (MMP-9) expression in A549 cells [23]. According to the only partial inhibition of expression and promoter activity of MMP-9 by PP1 in the respective study [23], the transactivation leading to MMP-9 expression was here also caused by the activation of a family of secreted and membrane-anchored MMPs. The latter induce the ectodomain cleavage of EGF ligands in the sense of the formation of biologically active soluble factors [47]. Others have shown that IL-1β in Caco-2 epithelial cells led to an upregulation of the EGFR ligand epiregulin via a JNK- but not Src-dependent pathway [27]. Further studies on oral squamous cell carcinoma cells have revealed that IL-1β mediates the formation of the chemokine CXCL1, which leads to a transactivation of EGFR via the G protein-coupled receptor CXCR2 [25]. 

In recent years, several studies have pointed to p38 [48,49,50] and p42/44 MAPKs [48,49] as well as JNK [48,50,51] as upstream regulators of TF expression. In accordance with a previous study of our group on human umbilical vein endothelial cells (HUVEC) [31], all kinases investigated in the present study (p42/44 MAPK, p38 MAPK, JNK) also showed participation in IL-1β-induced TF expression in A549 cells. Thereby, p38 MAPK and JNK proved to be EGFR-independent targets of IL-1β. In the case of p38 MAPK, analogous to our data, the simultaneous involvement of EGFR-independent p38 MAPK and EGFR-dependent p42/44 MAPK activation in renal cell death induced by reactive oxygen species could be demonstrated [52]. On the other hand, p38 MAPK was identified as a downstream target of EGFR in the regulation of cyclooxygenase-2 expression in pancreatic cancer cells [53]. Regarding JNK, one work described EGFR-dependent activation of the JNK signaling pathway in the B cell line DT-40 [54]. In addition, TF expression in glioblastoma cells mediated by EGFR overexpression or EGF stimulation was JNK1 dependent [15]. In summary, these exemplary results indicate a cell type-dependent activation of p38 MAPK and JNK after EGFR activation. A further explanation for this apparent discrepancy could also lie in the stimulus-dependent different tyrosine phosphorylation profiles at EGFR, which probably influence the subsequent differential kinetics of MAPK activation [52]. Interestingly, the EGFR knockdown in our experiments led to an upregulation of JNK activation, which could be interpreted as a possible compensation of the blocked EGFR/p42/44 MAPK pathway. Finally, of the numerous investigations demonstrating the EGFR-induced downstream activation of p42/44 MAPK, only two studies on IL-1β-induced EGFR transactivation should be mentioned here [21,26].

The involvement of both EGFR-independent and EGFR-dependent signaling pathways in IL-1β-induced TF expression is also supported by the fact that the EGFR TKIs erlotinib, gefitinib and afatinib—with complete inhibition of EGFR and p42/44 MAPK activation demonstrated for erlotinib—led to only partial inhibition of TF formation. The above compounds reversibly compete with ATP for the ATP binding site at the tyrosine kinase domain of EGFR, thereby inhibiting several EGF-triggered cancer-promoting signaling pathways [55,56]. All three EGFR TKIs are widely used to treat advanced NSCLC with EGFR mutations and have demonstrated their efficacy here [57]. Therefore, with regard to TF modulation, future studies should also focus on lung cancer cells with mutated, overactive EGFR. 

There are some points that deserve special remarks. First, it should be mentioned that PP1 and erlotinib also suppressed basal EGFR phosphorylation in our work. In addition, in both cases, as well as when cells were transfected with EGFR siRNA, the inhibition of basal p42/44 MAPK phosphorylation was detected. The causes of this apparently basal EGFR/p42/44 MAPK axis deserve further investigation. However, a relationship between the effects of the aforementioned inhibitors on basal and IL-1β-induced parameters cannot be deduced from these findings. Thus, in the case of PP1, the inhibition of IL-1β-induced EGFR activation was much more pronounced than the inhibition of basal EGFR activation, supporting the interference of this inhibitor with IL-1β action. Most crucially, however, a causal relationship between IL-1β-induced EGFR and subsequent p42/44 MAPK activation was clearly demonstrated using EGFR siRNA. Second, further studies are needed on the exact mechanism of TF induction by p38 MAPK and JNK in A549 cells. A potential target would be the transcription factor activator protein-1 (AP-1) involved in TF expression [58]. In a previous work on human monocytes, protease inhibitors were shown to induce TF expression via phosphorylation of p38 MAPK and JNK, followed by phosphorylation and the activation of AP-1 [59].

Last but not least, our data on the principal role of EGFR in TF expression are consistent with corresponding studies of other designs in glioma [14,15,16], epidermoid [14,17,18], colorectal [18], breast [19] and endometrial cancer cells [20]. However, to the best of our knowledge, erlotinib-induced inhibition of TF formation has before only been described in the case of TF upregulation in breast cancer cells induced by the p42/44 MAPK activation inhibitor PD98059 [19]. Here we provide the first evidence for EGFR TKIs to interfere with IL-1β-induced TF formation.

## 4. Materials and Methods

### 4.1. Materials

IL-1β human, IL-1Ra and PP1 were obtained from Sigma-Aldrich Corporation (Taufkirchen, Germany). Erlotinib hydrochloride, gefitinib and afatinib were bought from Santa Cruz Biotechnology, Inc. (Heidelberg, Germany). PD98059, Gibco^TM^ penicillin-streptomycin and Gibco^TM^ trypsin-EDTA were purchased from Thermo Fisher Scientific Inc. (Schwerte, Germany). SP600125 and SB203580 were from Tocris Bioscience (Wiesbaden-Nordenstadt, Germany). Dimethyl sulfoxide (DMSO), ethylenediaminetetraacetic acid (EDTA), glycerin, glycine, hydrochloric acid 37% (HCl), sodium chloride (NaCl), sodium hydroxide (NaOH), sodium dodecyl sulfate (SDS) ultra pure, Tris ultrapure and Tris hydrochloride (Tris HCl) were obtained from AppliChem (Darmstadt, Germany). 4-(2-Hydroxyethyl)-1-piperazineethanesulfonic acid (HEPES) and β-mercaptoethanol were purchased from Ferak Berlin GmbH (Berlin, Germany). Aprotinin, bromphenol blue, hydrogen peroxide solution (H_2_O_2_, 30%), luminol, orthovanadate, p-coumaric acid and phenylmethanesulfonyl fluoride (PMSF) were bought from Sigma-Aldrich Corporation. Leupeptin was from Biomol (Hamburg, Germany). Acrylamide (Rotiphorese^®^ Gel, 30%), albumin (IgG-free), ammonium peroxydisulphate (APS), acetic acid, *N*,*N*,*N*′,*N*′-tetramethylethylenediamine (TEMED) and Triton^®^ X-100, Tween^®^ 20 were purchased from Carl Roth GmbH + Co. KG (Karlsruhe, Germany). Methanol was bought from J. T. Baker (Griesheim, Germany). Aqua ad iniectabilia was from Braun Melsungen AG (Melsungen, Germany). Dulbecco’s modified eagle medium (DMEM) with 4.5 g/L glucose and with UltraGlutamine^TM^ I was bought from Lonza Cologne GmbH (Cologne, Germany). Dulbecco’s phosphate-buffered saline (DPBS) and fetal bovine serum (FBS) were purchased from PAN-Biotech GmbH (Aidenach, Germany). Milk powder was obtained from Bio-Rad Laboratories GmbH (Munich, Germany).

### 4.2. Cell Culture

A549 human lung carcinoma cells were purchased from DSMZ (Deutsche Sammlung von Mikroorganismen und Zellkulturen GmbH, Braunschweig, Germany; DSMZ no.: ACC 107, RRID:CVCL_0023). Species confirmation as human was carried out by the supplier using isoelectric focusing of malate dehydrogenase, nucleosid phosphorylase and fingerprint. Multiplex PCR of minisatellite markers revealed a unique DNA profile. Cells were frozen in large stock at early passages and were used within 6 months following resuscitation. 

A549 lung carcinoma cells were cultured in DMEM supplemented with 10% heat-inactivated FBS, 100 U/mL penicillin and 100 μg/mL streptomycin. The cells were grown in a humidified incubator at 37 °C and 5% CO_2_. All incubations with test substances were performed in serum-free DMEM, after washing cells with DPBS. The test substances were dissolved in DPBS (IL-1β, IL-1Ra) or DMSO (PP1, erlotinib, gefitinib, afatinib, PD98059, SP600125, SB203580), the latter with a final concentration of 0.1% (*v*/*v*) in incubates. If used, the vehicle controls contained the appropriate concentration of DMSO. In all experiments, incubation media of vehicle- and substance-treated cells contained the same amount of solvents.

### 4.3. Determination of TF Protein

A549 cells were seeded on 6-well plates with a density of 200,000 cells per well in serum-containing DMEM and cultivated for 24 h. Before starting incubation with test substances, the cells were washed with DPBS and starved in serum-free DMEM for 12 h. The cells were then incubated with test substances or vehicles in serum-free DMEM for the specified periods. The supernatants were subsequently aspirated, and the cells were lysed in Cell Lysis Buffer 1 from R&D Systems (Wiesbaden-Nordenstadt, Germany) for 30 min under shaking conditions (500 rpm, room temperature) and then centrifuged for 5 min (20,817× *g*, 4 °C). For further measurements supernatants were used. The Human Coagulation Factor III/Tissue Factor Quantikine^®^ ELISA from R&D Systems was used for the quantification of the TF protein according to the manufacturer’s instructions. The TF protein was normalized post hoc to the total protein amount measured with the Pierce™ BCA (bicinchoninic acid) Protein Assay Kit from Thermo Fisher Scientific Inc., as specified by the manufacturer.

### 4.4. Western Blot Analysis

For Western blot analyses, A549 cells were seeded in 6-well plates at a density of 200,000 cells per well and cultivated in DMEM with 10% heat-inactivated FBS for 24 h, with the exception of Figure 2A, where 1.2 × 10^6^ cells were seeded per Petri dish. The cells were then starved in serum-free DMEM for 12 h prior to the specified treatment.

In all analyses, except those for Src, cells were washed following the removal of supernatants, lysed in solubilization buffer (50 mM HEPES, 150 mM NaCl, 1 mM EDTA, 1% [*v*/*v*] Triton^®^ X-100, 10% [*v*/*v*] glycerol, 5.2 µL/mL aprotinin, 2 µL/mL leupeptin, 10 µL/mL orthovanadate, 10 µL/mL PMSF) for 30 min on ice under occasional vortexes and then centrifuged for 5 min (20,817× *g*, 4 °C). The resulting supernatants were collected for further protein analyses. 

In case of Src analysis, the cells were washed with ice-chilled PBS, and after scraping the cells in lysis buffer (2% [*w*/*v*] SDS, 40% [*v*/*v*] H_2_O, 10% [*v*/*v*] glycerol, 50% [*v*/*v*] 125 mM Tris-HCl [pH 6.8]), the proteins were denatured immediately at 95 °C with continuous shaking for 10 min. Subsequently, cells were centrifuged for 5 min (20,817× *g*, 4 °C), and the resulting supernatants were collected for further protein analyses.

The total protein in cell lysates was quantified using the Pierce™ BCA Protein Assay Kit. Equal amounts of proteins were separated on a 10% SDS-polyacrylamide gel, transferred to a nitrocellulose membrane and finally incubated with primary antibodies in Tris-buffered saline containing 5% (*w*/*v*) albumin and 0.1% (*v*/*v*) Tween^®^ 20 (TBS-T buffer) overnight at 4 °C. Specific antibodies raised against phospho-p44/42 MAPK (Erk1/2) (Thr202/Tyr204; #9101), p44/42 MAPK (Erk1/2; #9102), phospho-SAPK/JNK (Thr183/Tyr185; #9251), SAPK/JNK (#9252), phospho-p38 MAPK (Thr180/Tyr182; #9211), p38 MAPK (#9212), phospho-EGFR (Tyr1068; #2234), phospho-EGFR (Tyr845; #2231), EGFR (C74B9; #2646), phospho-Src family (Tyr416; #6943) and Src (36D10; #2109) were obtained from Cell Signaling Technology (Frankfurt/Main, Germany). The monoclonal β-actin antibody (clone AC-15, produced in mouse, ascites fluid, #A5441) was from Sigma-Aldrich Corporation. Following washing with TBS-T, the membranes were then incubated with horseradish peroxidase-bound secondary antibodies (anti-rabbit antibody, #7074; anti-mouse antibody, #7076; Cell Signaling Technology) in 1% (*w*/*v*) albumin-containing TBS-T buffer for 1 h at room temperature. Antibody binding was visualized by a chemiluminescent solution (100 mM Tris hydrochloride pH 8.5, 125 mM luminol, 200 mM p-coumaric acid, 0.09% [*v*/*v*] H_2_O_2_, 0.0072% [*v*/*v*] DMSO). The densitometric analysis of the band intensities was achieved by optical scanning and quantification with the 1-D analysis software Quantity One from Bio-Rad Laboratories GmbH (Munich, Germany). For band size identification, Precision Plus Protein™ Dual Color Standards from Bio-Rad Laboratories GmbH (Munich, Germany) were used. The phosphorylated forms were then normalized to the respective total form and the expression of the proteins was compared with that of the corresponding vehicle control. To document an even protein load in Western blots of cell lysates, the membranes were also examined with an antibody directed against β-actin.

### 4.5. Quantitative Reverse Transcriptase Polymerase Chain Reaction (RT-PCR)

A549 cells were cultivated after seeding in 24-well plates with a density of 100,000 cells per well for 24 h and then starved in serum-free DMEM for 12 h before the indicated treatment. Total RNA was isolated using the RNeasy Total RNA Kit from Qiagen GmbH (Hilden, Germany). Applied Biosystems^®^ TaqMan^®^ RNA-to-CT™ 1-Step Kit and Applied Biosystems^®^ TaqMan^®^ Gene Expression Assays, both obtained from Thermo Fisher Scientific Inc., were used for the quantification of TF (Assay ID: Hs01076029_m1, gene symbol: F3) and β-actin (Assay ID: Hs99999903_m1, gene symbol: ACTB) mRNA expression according to the manufacturer’s instructions. TF mRNA levels were normalized to β-actin mRNA and compared to the corresponding vehicle controls.

### 4.6. SiRNA Transfections

Transfection of EGFR siRNA or negative control siRNA was performed using the transfection reagent Lipofectamine™ RNAiMAX and the transfection medium Opti-MEM^®^ I Reduced Serum Medium, both obtained from Thermo Fisher Scientific Inc., according to the manufacturer’s instructions.

EGFR siRNA was purchased from Thermo Fisher Scientific Inc. (#1299001; siRNA HSS103116), and the negative control siRNA from Qiagen GmbH (#1022076). First of all, Opti-MEM^®^ I Reduced Serum Medium, Lipofectamine™ RNAiMAX transfection reagent and EGFR siRNA or negative control siRNA was added into 6-well plates. After 20 min, A549 cells were seeded at a density of 200,000 cells per well and grown for 24 h. Final concentrations of EGFR siRNA or negative control siRNA in incubates were 10 nM. Subsequently, cells were washed and incubated with test substances or vehicles for the indicated time periods. Finally, cells were harvested for further analyses, as previously described.

### 4.7. Cellular Viability Assay

Possible cytotoxicity of the tested compounds was excluded by viability assays with MTT (3-(4,5-Dimethyl-2-thiazolyl)-2,5-diphenyl-2*H*-tetrazolium bromide; Sigma-Aldrich Corporation, #M2128) using the incubation and time regimes employed in Figure 1C,D, Figure 4C–E and Figure 5E. To this end, A549 cells were seeded on 96-well plates at a density of 5000 cells per well in serum-containing DMEM and cultured for 24 h. Cells were then washed with DPBS, starved in serum-free DMEM for 12 h and subsequently incubated with test substances. After incubation, MTT at a final concentration of 0.5 mg/mL (*w*/*v*) was added to each well, and the cells were incubated for a further 2 h. The supernatants were then removed, 100 µL DMSO was added to each well and formazan formation was determined by measuring the absorbance at 570 nm (wavelength correction at 690 nm) with a microplate reader.

### 4.8. Statistics

Comparisons between the two groups were carried out using Student’s unpaired two-tailed *t* test. Comparisons between more than two groups were performed by one-way ANOVA with Bonferroni’s or Dunnett’s post hoc test. In the case of Bonferroni’s post hoc test, the determination of statistical significance was limited to the groups of interest for reasons of clarity of presentation. All statistical analyses were conducted with GraphPad Prism 7.02 (GraphPad Software, Inc., San Diego, CA, USA).

## 5. Conclusions

A detailed understanding of the molecular mechanisms of TF expression in cancer cells is of enormous importance for the development of individual cancer treatment strategies and combination therapies. Against this background, concepts directed at already formed TF, such as anti-TF antibody-drug conjugates, TF/FVIIa targeting agents and inhibitors of TF-mediated PAR signaling, are already being tested clinically and preclinically [6,7]. Another strategy could be the inhibition of TF expression. In this context, the present study demonstrates a previously unknown dual IL-1β-triggered mechanism of TF expression in lung cancer cells and thus defines upstream targets for pharmacotherapeutic intervention to block the formation of protumorigenic TF. The proven inhibition of IL-1β-induced TF expression by various EGFR TKIs and IL-1Ra underlines the fact that a corresponding effect could also be part of the action of already approved drugs.

## Data Availability

Data are available upon reasonable request.

## References

[1] Nemerson Y., Pitlick F.A. (1970). Purification and characterization of the protein component of tissue factor. Biochemistry.

[2] Bjorklid E., Storm E. (1977). Purification and some properties of the protein component of tissue thromboplastin from human brain. Biochem. J..

[3] Harlos K., Martin D.M., O’Brien D.P., Jones E.Y., Stuart D.I., Polikarpov I., Miller A., Tuddenham E.G., Boys C.W. (1994). Crystal structure of the extracellular region of human tissue factor. Nature.

[4] Rak J., Milsom C., Yu J. (2008). Tissue factor in cancer. Curr. Opin. Hematol..

[5] Leppert U., Eisenreich A. (2015). The role of tissue factor isoforms in cancer biology. Int. J. Cancer.

[6] Van Dreden P., Εlalamy Ι., Gerotziafas G.T. (2017). The Role of Tissue Factor in Cancer-Related Hypercoagulability, Tumor Growth, Angiogenesis and Metastasis and Future Therapeutic Strategies. Crit. Rev. Oncog..

[7] Hisada Y., Mackman N. (2019). Tissue Factor and Cancer: Regulation, Tumor Growth, and Metastasis. Semin. Thromb. Hemost..

[8] Jiang X., Bailly M.A., Panetti T.S., Cappello M., Konigsberg W.H., Bromberg M.E. (2004). Formation of tissue factor-factor VIIa-factor Xa complex promotes cellular signaling and migration of human breast cancer cells. J. Thromb. Haemost..

[9] Hu L., Xia L., Zhou H., Wu B., Mu Y., Wu Y., Yan J. (2013). TF/FVIIa/PAR2 promotes cell proliferation and migration via PKCα and ERK-dependent c-Jun/AP-1 pathway in colon cancer cell line SW620. Tumour. Biol..

[10] Koomägi R., Volm M. (1998). Tissue-factor expression in human non-small-cell lung carcinoma measured by immunohistochemistry: Correlation between tissue factor and angiogenesis. Int. J. Cancer.

[11] Goldin-Lang P., Tran Q.V., Fichtner I., Eisenreich A., Antoniak S., Schulze K., Coupland S.E., Poller W., Schultheiss H.P., Rauch U. (2008). Tissue factor expression pattern in human non-small cell lung cancer tissues indicate increased blood thrombogenicity and tumor metastasis. Oncol. Rep..

[12] Sawada M., Miyake S., Ohdama S., Matsubara O., Masuda S., Yakumaru K., Yoshizawa Y. (1999). Expression of tissue factor in non-small-cell lung cancers and its relationship to metastasis. Br. J. Cancer.

[13] Regina S., Valentin J.B., Lachot S., Lemarié E., Rollin J., Gruel Y. (2009). Increased tissue factor expression is associated with reduced survival in non-small cell lung cancer and with mutations of TP53 and PTEN. Clin. Chem..

[14] Milsom C.C., Yu J.L., Mackman N., Micallef J., Anderson G.M., Guha A., Rak J.W. (2008). Tissue factor regulation by epidermal growth factor receptor and epithelial-to-mesenchymal transitions: Effect on tumor initiation and angiogenesis. Cancer Res..

[15] Rong Y., Belozerov V.E., Tucker-Burden C., Chen G., Durden D.L., Olson J.J., Van Meir E.G., Mackman N., Brat D.J. (2009). Epidermal growth factor receptor and PTEN modulate tissue factor expression in glioblastoma through JunD/activator protein-1 transcriptional activity. Cancer Res..

[16] Magnus N., Garnier D., Rak J. (2010). Oncogenic epidermal growth factor receptor up-regulates multiple elements of the tissue factor signaling pathway in human glioma cells. Blood.

[17] Yu J.L., Xing R., Milsom C., Rak J. (2010). Modulation of the oncogene-dependent tissue factor expression by kinase suppressor of ras 1. Thromb. Res..

[18] Garnier D., Magnus N., Lee T.H., Bentley V., Meehan B., Milsom C., Montermini L., Kislinger T., Rak J. (2012). Cancer cells induced to express mesenchymal phenotype release exosome-like extracellular vesicles carrying tissue factor. J. Biol. Chem..

[19] Hu C., Huang L., Gest C., Xi X., Janin A., Soria C., Li H., Lu H. (2012). Opposite regulation by PI3K/Akt and MAPK/ERK pathways of tissue factor expression, cell-associated procoagulant activity and invasiveness in MDA-MB-231 cells. J. Hematol. Oncol..

[20] Kato S., Pinto M., Carvajal A., Espinoza N., Monsó C., Bravo L., Villalon M., Cuello M., Quest A.F., Suenaga A. (2005). Tissue factor is regulated by epidermal growth factor in normal and malignant human endometrial epithelial cells. Thromb. Haemost..

[21] Wan Y., Belt A., Wang Z., Voorhees J., Fisher G. (2001). Transmodulation of epidermal growth factor receptor mediates IL-1β-induced MMP-1 expression in cultured human keratinocytes. Int. J. Mol. Med..

[22] Tanida S., Joh T., Itoh K., Kataoka H., Sasaki M., Ohara H., Nakazawa T., Nomura T., Kinugasa Y., Ohmoto H. (2004). The mechanism of cleavage of EGFR ligands induced by inflammatory cytokines in gastric cancer cells. Gastroenterology.

[23] Cheng C.Y., Kuo C.T., Lin C.C., Hsieh H.L., Yang C.M. (2010). IL-1β induces expression of matrix metalloproteinase-9 and cell migration via a c-Src-dependent, growth factor receptor transactivation in A549 cells. Br. J. Pharmacol..

[24] Sanchez-Guerrero E., Chen E., Kockx M., An S.W., Chong B.H., Khachigian L.M. (2012). IL-1beta signals through the EGF receptor and activates Egr-1 through MMP-ADAM. PLoS ONE.

[25] Lee C.H., Syu S.H., Liu K.J., Chu P.Y., Yang W.C., Lin P., Shieh W.Y. (2015). Interleukin-1 beta transactivates epidermal growth factor receptor via the CXCL1-CXCR2 axis in oral cancer. Oncotarget.

[26] Broekman W., Amatngalim G.D., de Mooij-Eijk Y., Oostendorp J., Roelofs H., Taube C., Stolk J., Hiemstra P.S. (2016). TNF-α and IL-1β-activated human mesenchymal stromal cells increase airway epithelial wound healing in vitro via activation of the epidermal growth factor receptor. Respir. Res..

[27] Massip-Copiz M., Clauzure M., Valdivieso Á.G., Santa-Coloma T.A. (2018). Epiregulin (EREG) is upregulated through an IL-1β autocrine loop in Caco-2 epithelial cells with reduced CFTR function. J. Cell. Biochem..

[28] Bevilacqua M.P., Pober J.S., Majeau G.R., Cotran R.S., Gimbrone M.A. (1984). Interleukin 1 (IL-1) induces biosynthesis and cell surface expression of procoagulant activity in human vascular endothelial cells. J. Exp. Med..

[29] Archipoff G., Beretz A., Freyssinet J.M., Klein-Soyer C., Brisson C., Cazenave J.P. (1991). Heterogeneous regulation of constitutive thrombomodulin or inducible tissue-factor activities on the surface of human saphenous-vein endothelial cells in culture following stimulation by interleukin-1, tumour necrosis factor, thrombin or phorbol ester. Biochem. J..

[30] Puhlmann M., Weinreich D.M., Farma J.M., Carroll N.M., Turner E.M., Alexander H.R. (2005). Interleukin-1β induced vascular permeability is dependent on induction of endothelial tissue factor (TF) activity. J. Transl. Med..

[31] Scholl A., Ivanov I., Hinz B. (2016). Inhibition of interleukin-1β-induced endothelial tissue factor expression by the synthetic cannabinoid WIN 55;212-2. Oncotarget.

[32] Osnes L.T., Westvik A.B., Joø G.B., Okkenhaug C., Kierulf P. (1996). Inhibition of IL-1 induced tissue factor (TF) synthesis and procoagulant activity (PCA) in purified human monocytes by IL-4, IL-10 and IL-13. Cytokine.

[33] Balkwill F., Mantovani A. (2001). Inflammation and cancer: Back to Virchow?. Lancet.

[34] De Vita F., Orditura M., Auriemma A., Infusino S., Catalano G. (1998). Serum concentrations of proinflammatory cytokines in advanced non small cell lung cancer patients. J. Exp. Clin. Cancer Res..

[35] Lin C.C., Kuo C.T., Cheng C.Y., Wu C.Y., Lee C.W., Hsieh H.L., Lee I.T., Yang C.M. (2009). IL-1β promotes A549 cell migration via MAPKs/AP-1- and NF-κB-dependent matrix metalloproteinase-9 expression. Cell Signal..

[36] Eda H., Burnette B.L., Shimada H., Hope H.R., Monahan J.B. (2011). Interleukin-1β-induced interleukin-6 production in A549 cells is mediated by both phosphatidylinositol 3-kinase and interleukin-1 receptor-associated kinase-4. Cell Biol. Int..

[37] Shah S., Mostafa M.M., McWhae A., Traves S.L., Newton R. (2016). Negative Feed-forward Control of Tumor Necrosis Factor (TNF) by Tristetraprolin (ZFP36) Is Limited by the Mitogen-activated Protein Kinase Phosphatase, Dual-specificity Phosphatase 1 (DUSP1): IMPLICATIONS FOR REGULATION BY GLUCOCORTICOIDS. J. Biol. Chem..

[38] Aarreberg L.D., Esser-Nobis K., Driscoll C., Shuvarikov A., Roby J.A., Gale M. (2019). Interleukin-1β Induces mtDNA Release to Activate Innate Immune Signaling via cGAS-STING. Mol. Cell..

[39] Kopetz S. (2007). Targeting SRC and epidermal growth factor receptor in colorectal cancer: Rationale and progress into the clinic. Gastrointest. Cancer Res..

[40] Chen Z., Oh D., Dubey A.K., Yao M., Yang B., Groves J.T., Sheetz M. (2018). EGFR family and Src family kinase interactions: Mechanics matters?. Curr. Opin. Cell. Biol..

[41] Daub H., Wallasch C., Lankenau A., Herrlich A., Ullrich A. (1997). Signal characteristics of G protein-transactivated EGF receptor. EMBO J..

[42] Buteau J., Foisy S., Joly E., Prentki M. (2003). Glucagon-like peptide 1 induces pancreatic β-cell proliferation via transactivation of the epidermal growth factor receptor. Diabetes.

[43] Zhuang S., Schnellmann R.G. (2004). H_2_O_2_-induced transactivation of EGF receptor requires Src and mediates ERK1/2, but not Akt, activation in renal cells. Am. J. Physiol. Renal Physiol..

[44] Biscardi J.S., Maa M.C., Tice D.A., Cox M.E., Leu T.H., Parsons S.J. (1999). c-Src-mediated phosphorylation of the epidermal growth factor receptor on Tyr845 and Tyr1101 is associated with modulation of receptor function. J. Biol. Chem..

[45] Amos S., Martin P.M., Polar G.A., Parsons S.J., Hussaini I.M. (2005). Phorbol 12-myristate 13-acetate induces epidermal growth factor receptor transactivation via protein kinase Cdelta/c-Src pathways in glioblastoma cells. J. Biol. Chem..

[46] Zhang J., Kalyankrishna S., Wislez M., Thilaganathan N., Saigal B., Wei W., Ma L., Wistuba I.I., Johnson F.M., Kurie J.M. (2007). SRC-family kinases are activated in non-small cell lung cancer and promote the survival of epidermal growth factor receptor-dependent cell lines. Am. J. Pathol..

[47] Gschwind A., Zwick E., Prenzel N., Leserer M., Ullrich A. (2001). Cell communication networks: Epidermal growth factor receptor transactivation as the paradigm for interreceptor signal transmission. Oncogene.

[48] Steffel J., Akhmedov A., Greutert H., Lüscher T.F., Tanner F.C. (2005). Histamine induces tissue factor expression: Implications for acute coronary syndromes. Circulation.

[49] Gebhard C., Breitenstein A., Akhmedov A., Gebhard C.E., Camici G.G., Lüscher T.F., Tanner F.C. (2010). Amphetamines induce tissue factor and impair tissue factor pathway inhibitor: Role of dopamine receptor type 4. Eur. Heart J..

[50] Ding L., Ma W., Littmann T., Camp R., Shen J. (2011). The P2Y_2_ nucleotide receptor mediates tissue factor expression in human coronary artery endothelial cells. J. Biol. Chem..

[51] Stähli B.E., Camici G.G., Steffel J., Akhmedov A., Shojaati K., Graber M., Lüscher T.F., Tanner F.C. (2006). Paclitaxel enhances thrombin-induced endothelial tissue factor expression via c-Jun terminal NH_2_ kinase activation. Circ. Res..

[52] Dong J., Ramachandiran S., Tikoo K., Jia Z., Lau S.S., Monks T.J. (2004). EGFR-independent activation of p38 MAPK and EGFR-dependent activation of ERK1/2 are required for ROS-induced renal cell death. Am. J. Physiol. Renal Physiol..

[53] Hu H., Han T., Zhuo M., Wu L.L., Yuan C., Wu L., Lei W., Jiao F., Wang L.W. (2017). Elevated COX-2 Expression Promotes Angiogenesis Through EGFR/p38-MAPK/Sp1-Dependent Signalling in Pancreatic Cancer. Sci. Rep..

[54] Hashimoto A., Kurosaki M., Gotoh N., Shibuya M., Kurosaki T. (1999). Shc regulates epidermal growth factor-induced activation of the JNK signaling pathway. J. Biol. Chem..

[55] Schettino C., Bareschino M.A., Ricci V., Ciardiello F. (2008). Erlotinib: An EGF receptor tyrosine kinase inhibitor in non-small-cell lung cancer treatment. Expert Rev. Respir. Med..

[56] Ko J.C., Ciou S.C., Jhan J.Y., Cheng C.M., Su Y.J., Chuang S.M., Lin S.T., Chang C.C., Lin Y.W. (2009). Roles of MKK1/2-ERK1/2 and phosphoinositide 3-kinase-AKT signaling pathways in erlotinib-induced Rad51 suppression and cytotoxicity in human non-small cell lung cancer cells. Mol. Cancer Res..

[57] Yang Z., Hackshaw A., Feng Q., Fu X., Zhang Y., Mao C., Tang J. (2017). Comparison of gefitinib, erlotinib and afatinib in non-small cell lung cancer: A meta-analysis. Int. J. Cancer.

[58] Mackman N. (1997). Regulation of the tissue factor gene. Thromb. Haemost..

[59] Ohsawa M., Koyama T., Nara N., Hirosawa S. (2003). Induction of tissue factor expression in human monocytic cells by protease inhibitors through activating activator protein-1 (AP-1) with phosphorylation of Jun-N-terminal kinase and p38. Thromb. Res..

